# Dandy-Walker Malformation with Neonatal Meningitis: A Case Report

**DOI:** 10.31729/jnma.8472

**Published:** 2024-02-29

**Authors:** Bishal Pradhan, Bishal Sharma, Pratistha Acharya, Suraksha Thapa, Jyoti Chand, Simran Bista

**Affiliations:** 1 Nepalese Army Institute of Health Sciences, Sanobharyang, Kathmandu, Nepal; 2 Department of Paediatrics, Shree Birendra Hospital, Chhauni, Kathmandu, Nepal; 3 Kathmandu Medical College and Teaching Hospital, Sinamangal, Kathmandu, Nepal

**Keywords:** *case reports*, *Dandy-Walker malformation*, *magnetic resonance imaging*, *meningitis*

## Abstract

Dandy-Walker syndrome is a rare congenital central nervous system malformation. Dandy-Walker variant is characterised by cerebellar vermian hypoplasia, cystic fourth ventricular dilatation, and normal posterior fossa volume. Various prenatal tests such as ultrasound, fetal magnetic resonance imaging, and amniocentesis can help diagnose Dandy-Walker syndrome. Here, we report a case of the Dandy-Walker variant with meningitis in a neonate admitted to the neonatal intensive care unit due to multiple petechiae on the anterior abdominal wall, accompanied by peripheral cyanosis at the time of birth. Although maximum cases are diagnosed prenatally, some cases might be missed due to inadequate antenatal examination. Magnetic imaging resonance of the brain is best for the diagnosis of Dandy-Walker syndrome postnatally.

## INTRODUCTION

Dandy-Walker Syndrome (DWS) is a rare congenital malformation of the central nervous system (CNS), characterised by agenesis or hypoplasia of the cerebellar vermis, cystic dilatation of the fourth ventricle and enlargement of the posterior fossa cistern.^1^ The prevalence of DWM and its variations is 1 in 35,000 live births. Dandy-Walker variant is characterised by cerebellar vermian hypoplasia, cystic fourth ventricular dilatation, and normal posterior fossa volume.^2^ Developmental delay, hypotonia or spasticity, ataxia, and hydrocephalus are the common signs of Dandy-Walker syndrome.^3^ Here, we report the case of the Dandy-Walker variant with meningitis in a neonate.

## CASE REPORT

We report a case of a male neonate delivered via lower segment caesarean section following a failed induction of labour at 41 weeks and 1 day of gestation. Apgar score was 6/10 and 7/10 at 1 and 5 minutes of life respectively. The baby cried only after stimulation. The initial cry was weak. The maternal profile was Gravida 3 abortion 1 living 1 (G3A1L1) with no known risk factors.

He was admitted to the neonatal intensive care unit (NICU) due to multiple petechiae on the anterior abdominal wall, accompanied by peripheral cyanosis. New petechiae were observed after the admission at NICU on the anterior trunk and thorax region.

Suspecting presumed early-onset neonatal sepsis antibiotics were administered. Septic screening was sent which was normal. The neonate developed tachypnea at NICU due to which he was kept under oxygen supplementation via a headbox. Due to persistent tachypnea, the neonate was kept under continuous positive airway pressure (CPAP) which was gradually weaned off at around the 5th day of life. In the meantime, an increase in the baby's head circumference by 3 cm (34 at the time of birth to 37 on the 6th day of life) was observed.

**Figure 1:**
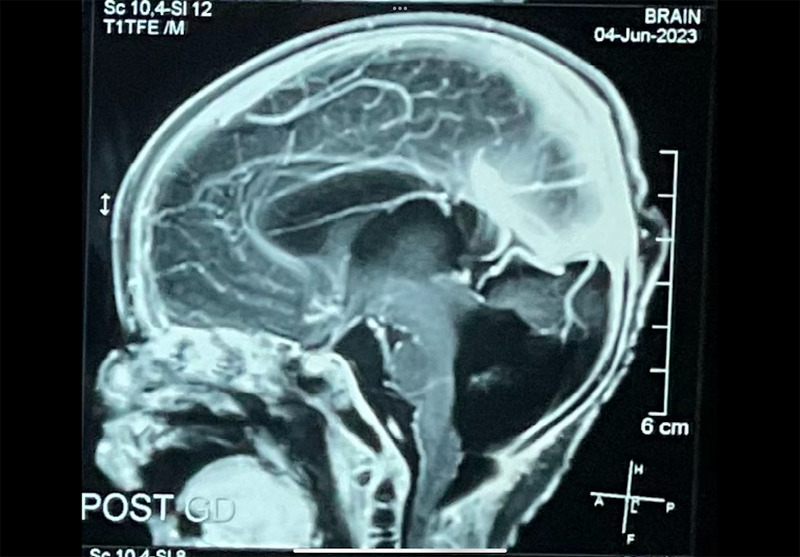
Cystic malformation of the posterior fossa.

A urine cytomegalovirus (CMV) polymerase chain reaction (PCR) test was done which was negative. Ultrasonography (USG) transcranium showed mild lateral ventricle dilatation with the prominence of the third and fourth ventricles. Mild hydrocephalus was detected in the USG. Sulcation, gyration, and corpus callosum were normal. Daily measurement of the occipitofrontal circumference was suggested. MRI of the brain was done which revealed hypoplastic cerebellar vermis with only the superior portion visualised. There was a cystic lesion in the posterior fossa which was in continuity with the third and fourth ventricle confirming the diagnosis of the Dandy Walker variant (Figure 1).

Increased meningeal vascularity was also detected, suggesting meningeal inflammation. Thus, a lumbar puncture was done which revealed a decreased glucose level (40.5 mg/dL) and elevated protein (56.9 mg/dL)level confirming meningitis. The CSF picture was normal, and no microorganisms were seen. On the twelfth day, an episode of abnormal posturing with the extension of all four limbs, head and spine stiffness was observed (generalized tonic-clonic seizure) which lasted for 30 seconds. Biochemical investigation was done which was normal.

Antibiotics cefotaxime and gentamicin were administered at early NICU admission for presumed early-onset neonatal sepsis. However, after keeping the baby under CPAP intravenous (IV) antibiotics were upgraded to meropenem and linezolid. For the seizure episodes, an injection of levetiracetam was added. Antibiotics were continued for 21 days, and levetiracetam was shifted to the oral form.

The case was consulted with the neurosurgery department where a ventriculoperitoneal (VP) shunt surgery was planned for Dandy-Walker syndrome. However, it was postponed at the time due to associated meningitis. The newborn was discharged on syrup levetiracetam and oral vitamin D supplement.

Vision and hearing evaluation was done in the follow-up which were normal. A test to rule out TORCH infection was also done which was negative. The thyroid function test was normal. The baby was consulted for physiotherapy for increased tone of the upper limb. The baby is still on levetiracetam and vitamin D supplementation.

## DISCUSSION

Dandy-Walker syndrome is a rare neurological condition that impairs the development of the cerebellum. The tentorium and torcula are displaced upward due to the posterior fossa anomaly, characterised by agenesis or hypoplasia of the vermis and cystic expansion of the fourth ventricle.^2^ In our case, the baby had hypoplastic cerebellar vermis with only the superior portion visualised. There was a cystic lesion in the posterior fossa which was in continuity with the third and fourth ventricle. However, the cystic expansion of the fourth ventricle had not developed, confirming the type of DandyWalker variant.

Modern neuroimaging has transformed the radiographic diagnosis of DWS. Pneumoencephalography and angiography are invasive procedures that have been replaced by computed tomography (CT) and more recently MRI.^4^ We also established the diagnosis of DWS with the help of an MRI scan. Most patients have hydrocephalus at the time of diagnosis of DWS.^5^ The patient in our case also had mild hydrocephalus revealed by transcranial USG. The finding was supported by MRI.

Various prenatal tests (ultrasound, fetal magnetic resonance imaging, and genetic testing) can help diagnose DWS. Dandy-Walker variant babies may have reduced cerebral fluid accumulation. Their harm to the cerebellar structures will be reduced as a consequence. Surgical intervention to drain excess fluid from the brain is less common in babies with this kind of DWS.^6^ The baby in our case is also under medication for seizures. Although the surgery VP shunting was planned at the time of diagnosis, it was postponed due to meningitis. In the follow-up as the baby is stable, he is continued on the same medication.

A ventriculoperitoneal shunt is usually done to drain excess cerebrospinal fluid (CSF) to prevent fluid accumulation which might increase the intracranial pressure. If the patient does not have an increasing head size in the follow-up, surgical intervention might not be needed. Treatment can include the management of problems and complications like seizures in our case.
